# Smart Wearables for Cardiac Monitoring—Real-World Use beyond Atrial Fibrillation

**DOI:** 10.3390/s21072539

**Published:** 2021-04-05

**Authors:** David Duncker, Wern Yew Ding, Susan Etheridge, Peter A. Noseworthy, Christian Veltmann, Xiaoxi Yao, T. Jared Bunch, Dhiraj Gupta

**Affiliations:** 1 Hannover Heart Rhythm Center, Department of Cardiology and Angiology, Hannover Medical School, 30625 Hannover, Germany; veltmann.christian@mh-hannover.de; 2 Liverpool Centre for Cardiovascular Science, Liverpool Heart and Chest Hospital, University of Liverpool, Liverpool L1 8JX, UK; dwyew@hotmail.com (W.Y.D.); dhiraj.gupta@lhch.nhs.uk (D.G.); 3 Department of Pediatrics, University of Utah, Salt Lake City, UT 84108, USA; Susan.Etheridge@hsc.utah.edu; 4 Robert D. and Patricia E. Kern Center for the Science of Health Care Delivery, Mayo Clinic, Rochester, MN 55902, USA; Noseworthy.Peter@mayo.edu (P.A.N.); Yao.Xiaoxi@mayo.edu (X.Y.); 5 Department of Cardiovascular Medicine, Mayo Clinic, Rochester, MN 55902, USA; 6 Department of Medicine, School of Medicine, University of Utah, Salt Lake City, UT 84108, USA; Jared.Bunch@hsc.utah.edu

**Keywords:** wearables, remote monitoring, cardiac monitoring, artificial intelligence, arrhythmia, heart failure, digital health, mobile health

## Abstract

The possibilities and implementation of wearable cardiac monitoring beyond atrial fibrillation are increasing continuously. This review focuses on the real-world use and evolution of these devices for other arrhythmias, cardiovascular diseases and some of their risk factors beyond atrial fibrillation. The management of nonatrial fibrillation arrhythmias represents a broad field of wearable technologies in cardiology using Holter, event recorder, electrocardiogram (ECG) patches, wristbands and textiles. Implementation in other patient cohorts, such as ST-elevation myocardial infarction (STEMI), heart failure or sleep apnea, is feasible and expanding. In addition to appropriate accuracy, clinical studies must address the validation of clinical pathways including the appropriate device and clinical decisions resulting from the surrogate assessed.

## 1. Introduction

Advances in wearable technologies have provided new opportunities for the diagnosis and management of cardiovascular diseases and their risk factors. Technologies that are commonplace in clinical situations, such as monitors of heart rate and rhythm and blood pressure, are now available directly to consumers.

Traditionally, when a wearable medical device was advocated by a healthcare provider and prescribed, their use was supported by evidence of effectiveness, reliability and safety. Ongoing scrutiny of these devices was, through the clinical lens of treatments and outcomes, prompted by device diagnostics as well as through the regulatory processes that reviewed these measures and the potential additive value for reimbursement.

Initially, medical devices that are marketed directly to consumers were largely for health and wellness tracking and fitness assessment. However, these devices and their biologic sensors have become increasingly more complex, and as a consequence, their potential utility to diagnose and prompt treatment for cardiovascular disease and its risk factors has increased. If a device is used to support a healthy lifestyle and monitor fitness or activity levels, it may not require the same regulatory review as a wearable medical device that is prescribed by a healthcare provider. However, if the data provided can be used to influence therapeutic decisions and medical decision making, then the regulatory environment can be much more extensive, and claims for use must be supported with data of safety, efficacy and value.

In the United States, the regulatory organization that oversees claims of efficacy and safety is the Food and Drug Administration (FDA). In the United Kingdom, this is the Medicines and Healthcare Products Regulatory Agency (MHRA); in the European Union, it is the European Medicines Agency (EMA). The FDA has published multiple guidelines (https://www.fda.gov/medical-devices/digital-health-center-excellence (accessed on 2 April 2021)) for products in which regulatory requirements will be enforced as well as for others deemed “low risk” in which they will not. In the United Kingdom, the National Institute for Health and Care Excellence (NICE) published a standards framework for digital technologies, which provides the requirements and metrics new technologies must meet in order for approval (https://www.nice.org.uk/about/what-we-do/our-programmes/evidence-standards-framework-for-digital-health-technologies (accessed on 2 April 2021)).

This market of direct-to-consumer healthcare is increasing substantially. In the United States, it already represents a USD 700 billion industry [1]. A direct-to-consumer approach remains attractive to industry as a means to reach a broad consumer base with fewer regulatory hurdles. The wearable industry will expand further with the rapid use and assimilation of digital health. As these devices are increasingly being offered and utilized, it has been difficult for healthcare providers to manage the data and recommend which, if any, tools should be used for the diagnosis and management of cardiovascular diseases and its risk factors.

Healthcare providers play a critical role in helping patients navigate their health and must adapt to these technologies. However, this role is dependent on some degree of understanding about the validity of these medical devices and their associated value and limitations. For example, for cardiac rhythm monitoring, patients often assume that the monitoring is continuous, as it appears this way in the displayed graphs or figures on their smartphone. Nonetheless, current direct-to-consumer cardiac monitors are not continuous and are for the surveillance or “spot checking” of arrhythmias and, at this time, do not replace the continuous monitors that are prescribed by a healthcare provider. Those devices that require patient activation will remain limited due to their under-detection of subclinical or minimally symptomatic arrhythmias. Additionally, the amount and quality of data recorded and stored with direct-to-consumer cardiac devices depend on the battery capacity, storage capabilities, size of the device and cost of the device.

Early use of direct-to-consumer cardiac wearables beyond fitness has been led by those devices that diagnose cardiac arrhythmias, in particular, atrial fibrillation (AF). This review will focus on the real-world use and evolution of these devices for other arrhythmias, other cardiovascular diseases and some of their risk factors beyond AF.

## 2. Non-AF Arrhythmia Detection

The conventional 12-lead electrocardiogram (ECG) provides an accurate assessment of cardiac arrhythmias. Nonetheless, it offers only a “snapshot” of electrical signals and misses arrhythmias that occur outside this period. Hence, continuous ECG monitoring systems have been developed to compensate for this limitation. Currently, Holter monitors are the mainstay device for the ambulatory detection of clinical arrhythmias. Nonetheless, these devices are impractical in terms of size and offer only a limited duration of continuous monitoring. For a longer duration of cardiac monitoring (≤30 days), external loop and event monitors (intermittent) are available. An alternative is an implantable loop recorder, which may last for up to three years, but this involves an invasive procedure with potential risks, albeit small. Overall, there are significant cost implications associated with these established devices, in terms of hardware, software and technician support. As a result, these traditional systems are not directly accessible by the general population.

### 2.1. Types of Cardiac Monitoring

**Figure 1:**
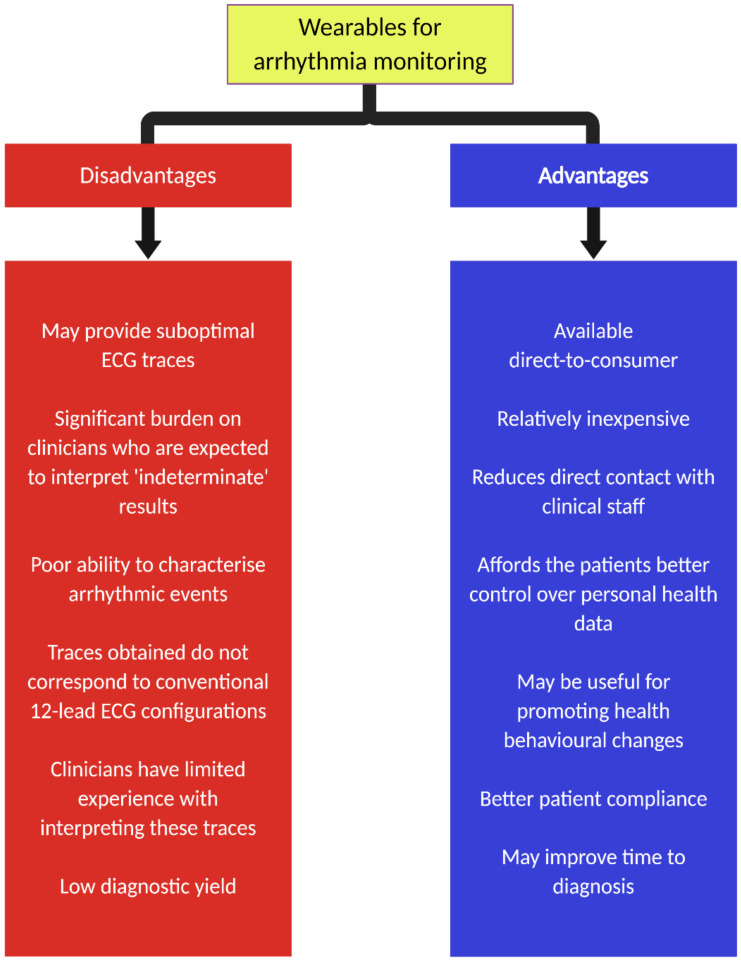
Generic advantages and disadvantages of wearables for monitoring of cardiac arrhythmia.

Over the past decade, there has been a surge in new wearables (e.g., patches and wristbands) that are capable of monitoring heart rate and ECG signals. The application of machine learning and artificial intelligence (AI) has further enhanced the role of these devices. Each offers a convenient, relatively affordable and directly accessible method of cardiac monitoring to the general public. Alongside an increase in consumers who are health-conscious or curious, these devices have been embraced with much enthusiasm. Table 1 summarizes different types of cardiac monitoring [2]. Advantages and disadvantages of wearables for arrhythmia monitoring are shown in Figure 1.

### 2.2. Mobile Cardiac Outpatient Telemetry

The advantages of these new wearables for the detection of cardiac arrhythmias have primarily been investigated in the context of AF, and stem largely from their longer duration of monitoring (i.e., detection of subclinical events). In the past, the use of a mobile cardiac outpatient telemetry (MCOT) system has been shown to provide a higher diagnostic yield than standard patient-activated external loop recorders (88% vs. 75%) among patients with symptoms suggestive of significant cardiac arrhythmia and a nondiagnostic result from 24-h Holter [3]. The MCOT system had a particular role in the detection of asymptomatic clinically significant arrhythmias [4], highlighting the benefits of continuous cardiac monitoring in at-risk patients. Newer MCOT systems, such as the NUVANT Mobile Cardiac Telemetry (Corventis, San Jose, CA, USA), comprise a wireless-enabled arrhythmia monitor that utilizes a low-profile PiiX sensor, lasting up to seven and a half days, to collect real-time ECG data. This device has shown promise in several settings [5,6] and been previously found to be associated with good compliance [7]. The use of these wireless-capable wearable devices enables remote physician monitoring and immediate action in the event of a serious cardiac arrhythmia, similar in many ways to modern day implantable pacemakers and defibrillators.

### 2.3. Ultraportable Electrocardiogram Patches

Another form of wearables that has gained popularity for the detection of arrhythmias is ultraportable ECG patches. An example is the Zio Patch (iRhythm Technologies, San Francisco, CA, USA) that provides a continuous single-lead ECG signal for up to 14 days without the need for battery replacement during this time. The device may also be activated during symptomatic episodes. The advantage of this device over traditional Holter systems is that it can be mailed directly to patients and self-applied. Once the monitoring period is over, it can be mailed back for analysis. It has recently been utilized to detect nonsustained ventricular tachycardia and premature ventricular contractions in a population-based research setting [8]. In a study of 146 patients, Barrett et al. demonstrated that the Zio Patch had a higher diagnostic yield for total arrhythmia detection (atrioventricular block, pause, supraventricular tachycardia, ventricular tachycardia and polymorphic ventricular tachycardia [and AF]) compared to Holter monitoring [9]. However, these results were largely driven by an imbalance in the monitoring duration (24 h with Holter vs. 14 days with Zio Patch). In fact, a secondary analysis of device performance over a simultaneous 24-h period found that the Holter monitor detected significantly more arrhythmic events compared to the Zio Patch. Nonetheless, 93.7% of patients in the study found the patch to be comfortable as opposed to 51.7% for the Holter monitor [9]. A cross-sectional study of 26,751 consecutive patients receiving the Zio Patch for clinical indications of cardiac monitoring demonstrated high patient compliance with a significant proportion of patients who had arrhythmias suffering from these events after the initial 48 h [10], thereby reinforcing the need for longer term monitoring with patient-tolerable devices.

Among patients presenting to the emergency department with symptoms suggestive of cardiac arrhythmia, but deemed suitable for outpatient ambulatory cardiac monitoring, application of the Zio Patch for up to 14 days was associated with a diagnostic yield of 63.2% for significant arrhythmias [11]. Similar application in patients with unexplained syncope who presented to the emergency department resulted in a diagnostic yield of 27.9% [12]. Conversely, the use of the Zio XT Patch in a community-based sample of older individuals led to relatively low detection rates of atrioventricular block (2nd degree Mobitz II or 3rd degree; 3%) and pauses of more than three seconds (3%) [13]. These results underscore the importance of patient selection in the overall diagnostic yield of wearable devices.

Other patch-based ECG monitoring systems have also been described for the detection of cardiac arrhythmias. For this purpose, the Wellysis S-Patch Cardio has been found to provide good quality ECG traces that enable an evaluation of supraventricular and ventricular arrhythmias [14]. Separately, the ATP100 patch has also been found to have comparable results to conventional ECG monitoring systems [15].

### 2.4. Delivery of Therapy with Wearables

Additional to the detection of cardiac arrhythmias, wearables may also have a role in delivering therapy. In this regard, wearable cardioverter-defibrillators (WCD) are designed to deliver shocks for potentially life-threatening ventricular arrhythmias. In the VEST trial, the use of the WCD (LifeVest, Zoll, Pittsburgh, PA, USA) in patients with acute myocardial infarction (MI) and severely impaired left ventricular function (ejection fraction ≤ 35%) led to 20 appropriate and 9 inappropriate shocks [16]. Despite this, there was no significant difference in the rate of arrhythmic death in the device group compared to the control group. The authors reported that the lack of survival benefit with the WCD may have been due to the low adherence to wearing the device. In real-world studies of the WCD, the rate of appropriate shock is 1.1–1.3% with an incidence of inappropriate shock of less than 1% [17,18,19]. Moreover, in some situations, the use of the device facilitated long-term care decisions regarding the need for implantable cardioverter-defibrillator (ICD) implantation.

### 2.5. Important Considerations

More recently, other forms of wearables with ECG monitoring capabilities have been designed. An example is the smart textile-based garments that have the ability to measure ECG from multiple locations [20,21,22]. These novel technologies may further expand this already rapidly developing field.

Although there are many clear advantages of longer ECG monitoring afforded by these direct-to-consumer wearables, there are some limitations worth considering. First, the quality of ECG traces is likely to be significantly reduced in the absence of medical supervision. Second, the application of these devices will invariably generate a significant amount of ECG traces that require interpretation. In many situations, automated algorithms with excellent accuracy in classifying single-lead ECG from wearables compared to standard 12-lead ECG [23] have been designed. However, there remains an issue with unclassified traces in as many as 33% of ECGs due to baseline artefact [24,25]. This may be reduced, to a degree, by the manual interpretation of the traces, but this also means a greater workload for healthcare professionals [24]. In the future, improvements in adaptive filters may help address this issue. Third, although these wearables may have a use for the initial detection of cardiac arrhythmias, it is important to note that they have a restricted role for the characterization of such events, particularly those relating to tachycardia. Fourth, the ECG traces acquired from these wearables do not correspond directly to those from conventional 12-lead ECG and Holter monitoring systems.

**Figure 2:**
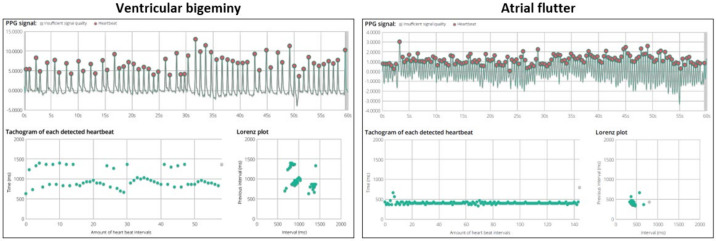
Examples of photoplethysmography (PPG) recordings for ventricular bigeminy and atrial flutter. The top graph shows PPG signals of a 60-s recording. The bottom-left tachogram displays all consecutive pulse signal intervals. For ventricular bigeminy, it can be seen that there are heart beats with alternating intervals. For atrial flutter, the heart rate is rapid with an average of 146 bpm. The Lorenz plot provides a visual representation for clustering patterns.

Additional to direct ECG monitoring systems, as described above, there have been efforts to correlate ECG traces with photoplethysmography (PPG) waveforms that rely on light transmission from smartphones to skin capillaries for the better detection of arrhythmias. Examples are shown in Figure 2.

For this purpose, each peak on the PPG signal is translated into an R wave which is then analyzed using prespecified algorithms. In the past, the Tateno-Glass method was described to test for the variation in R-R intervals [26,27]. Nevertheless, this method relied on the use of training data, which are often not representative of specific cohorts. Hence, newer algorithms that overcome this limitation may be preferred. More recently, Dash et al. described an algorithm that combines three statistical techniques: Root Mean Square of Successive R-R Differences (quantify variability), Turning Points Ratio (test for randomness of the time series) and Shannon entropy (characterize complexity) [28]. Other algorithms include the support vector machine, artificial neural network and k-nearest neighbor [29,30,31].

However, most of these algorithms were based on explicit features of peak-to-peak intervals with loss of information, such as amplitudes or waveforms, which decreases their accuracy in the presence of a high burden of premature atrial complexes (PACs). In this regard, deep learning-based algorithms that utilize all training data may perform better, as they are able to analyze characteristics such as amplitude, frequency and wave morphology [29].

Though most studies on PPG performance have focused on the detection of AF, it may also have a role in screening for other arrhythmias. In a small study of 20 participants comparing the use of a wrist wearable (Philips Cardio and Motion Monitoring Module) equipped with integrated optical PPG and accelerometer sensors against a 12-lead Holter, the former was able to identify episodes of bradycardia and tachycardia with a sensitivity of 85.0% and 99.4%, and specificity of 89.1% and 99.1%, respectively [32]. Elsewhere, Corino et al. used the Empatica E4 wristband and developed a classifier machine learning software that correctly identified arrhythmias, including atrial flutter, atrial tachycardia and premature ventricular contractions, with a sensitivity, specificity and accuracy of 75.8%, 76.8% and 80%, respectively. Furthermore, it was able to discriminate these arrhythmias from AF with a false negative rate of about 25% [33].

These PPG-based systems represent a potentially exciting avenue for more convenient ECG monitoring. Contactless rhythm analysis of facial PPG signals has even been described, which can be performed for several individuals simultaneously [34,35]. However, there are several constraints with this system. Overall, the PPG signals are highly vulnerable to artefacts that make traces uninterpretable, resulting in discarded data [36]. Furthermore, darker skin pigmentation and ambulation are factors that have been shown to reduce the accuracy of PPG-based devices, due to the modulation of the light wavelength by melanin and decreased device-to-skin contact [23,37]. The interpretability of black-box algorithms may also present a challenge for physicians who are expected to diagnose the results of these wearables.

### 2.6. Heart Rate Variability Analysis and QT Measurement

Other applications of wearables beyond the detection of cardiac arrhythmias are heart rate variability (HRV) analysis and QTc measurement. Pulse rate variability detected using PPG has been shown to be highly correlated with HRV measurements and, therefore, may be an important tool for the assessment of the autonomic nervous system and cardiovascular risk profile [38]. In a preliminary study of a wearable armband ECG monitor, Lazaro et al. found excellent results for HRV analysis using this new device compared to conventional Holter monitoring [39]. An advantage of this armband monitor over patch-based devices is that it does not utilize hydrogels, which may cause skin irritation. The measurement of QTc is an important aspect of risk assessment in specific populations and particularly in patients who are commencing certain medications with known QT prolonging effects. In a study of 36 patients (20 long QT syndrome [LQTS]; 16 controls), Castelletti et al. reported that the use of a remote monitoring ECG system with automated QTc measurement (BodyGuardian) was able to reliably identify prolonged QT intervals in comparison to a standard 12-lead Holter monitoring system (Mortara HScribe) [40]. Despite this, 34% of measurements had a disagreement of more than 20 ms, suggesting that further refinement is needed.

Overall, there are significant implications to contemplate with the use of wearables for the detection of cardiac arrhythmias (and other cardiovascular indices). Perhaps most importantly, there is a paucity of robust data comparing the accuracy, clinical implications and cost of these new wearables to established ECG systems. There may be a role for these devices as a screening rather than diagnostic tool. However, this approach will likely be associated with a low positive predictive value, which will invariably lead to increased levels of anxiety in people and unnecessary downstream testing in an otherwise largely asymptomatic population. Furthermore, the potential impact on healthcare resources (e.g., unwarranted visits to the emergency department) should not be underestimated. Moreover, the uptake of these wearables is mainly among the younger population who are inherently less likely to suffer from serious clinical arrhythmias, unlike their older counterparts, who may derive more benefits but may be reluctant to adopt these new technologies. Overall, the widespread, unregulated adoption of these new untested wearables for the detection of arrhythmias in an unselected population is unlikely to be beneficial. Instead, it may have a role in the long-term screening of clinically significant arrhythmias among high-risk patients.

## 3. ST-Segment Elevation Myocardial Infarction

An ST-segment elevation myocardial infarction (STEMI) is an abrupt severe manifestation of coronary artery disease associated with high morbidity and mortality, particularly if not diagnosed and treated rapidly. Ischemic heart disease is and will remain a significant contributor to death and disability worldwide [41]. In the United States, 550,000 first episodes of MI occur each year, and another 200,000 recurrent episodes occur [42]. As early diagnosis and management remain critical links in the outcomes related to STEMI, the broad availability and use of wearables and smartphones may help improve outcomes.

The diagnosis of an STEMI is based upon the presence of ST-elevation on a 12-lead electrocardiogram (ECG). ECG machines are available worldwide, but typically within the confines of healthcare resources, such as hospitals and clinics. However, when diagnosis can be made accurately, with the remote assistance of specialized care, appropriate treatment can be directed quickly and favorable outcomes of an early response maintained [43].

The majority of smartphone and smartwatch ECGs provide single lead telemetry and have been used for arrhythmia detection. A single lead is not ideal for the detection of arrhythmia, but much worse for ischemic disease in which leads outside of the territory of injury may show no significant ECG findings. Dynamic use of a smartwatch beyond the traditional configuration can replicate the Einthoven (leads I, II and III), and pseudo-unipolar chest leads to replicating a 6-lead ECG [44]. However, the nuances of segmental samples using a traditional smartwatch to obtain a 6-lead ECG make this approach limited for the diagnosis of STEMI.

**Figure 3:**
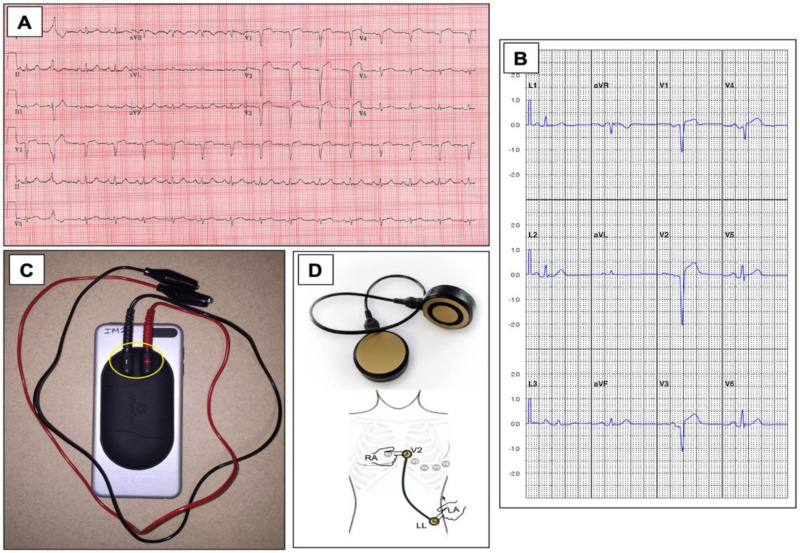
The figure shows a standard ECG (**A**) and a compared “12-lead equivalent ECG” (**B**) from the same patient using 2 electrodes connected to a smartphone associated sensor as shown (**C**) that obtains multiple sequential single-lead ECG measurements that was used in the St. Leuis trial. A next-generation concept for improved detection from AliveCor (Mountainview, CA, USA) is shown in (**D**) for smart phone-based cordless detection of STEMI with direct lead equivalents annotated. (Figures (**A**–**C**) are courtesy of Dr. Brent Muhlestein and Viet Le. Figure (**D**) is courtesy of Dr. Dave Alpert).

A standard 12-lead ECG requires the use of 9 electrodes that are strategically placed on the chest and limbs. Recently, a system (AliveCor, Mountainview, CA, USA) that uses 2 electrodes on a smartphone-associated sensor that obtains sequential single-lead ECG measurements that are averaged, uses a single limb lead as the ground for the precordial leads and requires vector summation of the multiple tracings was proposed. The system then creates what has been labelled as an “12-lead equivalent ECG”, as shown in Figure 3.

The accuracy of the smartphone-based 12-lead equivalent ECG was measured against a standard 12-lead ECG in 200 consenting patients who presented with acute chest pain in the ST-Leuis trial [45]. The smartphone 12-lead equivalent ECG quality was graded as good in 151 (74.0%), fair in 32 (15.7%), poor in 8 (3.9%) and uninterpretable in 13 (6.4%) of the patients. An STEMI or STEMI-equivalent diagnosis (new left bundle branch block, etc) was identified by a standard 12-lead ECG in 57/204 (27.9%) of the analyzed ECGs. The sensitivity, specificity and positive and negative predictive values for STEMI and STEMI using the 12-lead equivalent ECG provided by the smartphone were 0.89, 0.84, 0.70 and 0.95, respectively, when compared to the standard ECG.

Although the results for the ST-Leuis feasibility study were promising, several key technologic issues were identified and can be used with technological refinements of the tool. First, the smartphone technique requires sequential measurements to be made, which produce tracings that are contemporaneous but nonsimultaneous, and this can introduce variance into the signal and opportunity for error. Next, augmented limb leads are a computer averaged summation of multiple tracings; if these tracings have artifact or signal contamination, then the negative impact to the signal can be magnified. The precordial leads are obtained using a single limb lead for ground, and a second lead for a group may improve signal quality and minimize failure risk. Finally, the tracings are presented as an averaged tracing, so no raw signal data are preserved to allow direct interpretation. To minimize morphological corruption by signal averaging, algorithms were employed to exclude arrhythmia, such as premature ventricular complexes from the averaging process; such exclusion may be problematic with the dynamic variation of morphology that can be seen with acute ischemia or in a state when frequent ectopy may be present.

In a small case series of 2 patients that presented with an STEMI, an Apple Watch 4 (Apple, Cupertino, CA, USA) was used to create a 3-lead ECG [46] as an alternative diagnostic method. The 3 leads obtained matched the corresponding 3 leads on a standard ECG to suggest the potential application of multisite sampling using a smartwatch for the early detection of myocardial injury.

As these technologies improve from an ease of use and technology perspective, or others emerge, it is very feasible that the broad early diagnosis of STEMI using smart devices will materialize. Critical to these advances is the need for healthcare systems to rapidly assimilate the data, understand their validity and accuracy and then use them to improve critical metrics, such as those needed for early reperfusion.

## 4. Heart Failure

### 4.1. Prevalence of Heart Failure in the Community (Known vs. Unknown)

Currently, around 64.3 million people suffer from heart failure worldwide, with this number generally increasing [47]. The main reasons behind this are linked to an aging, global population growth and better survival under contemporary heart failure therapy. Heart failure prevalence differs depending on geographic region and socio-economic status. In developed countries, the prevalence of heart failure varies between 0.8% and 4.0% of the adult population with an increasing incidence with increasing age [48]. This wide range occurs as a result of the variety of methods used to estimate heart failure prevalence. Prevalence measures rely on different models and estimations based on the International Statistical Classification of Diseases and Related Health Problems-codes, patients’ clinical signs and symptoms, echocardiographic exams and natriuretic peptides, which each have their own limitations. This is further confounded by the fact that half of heart failure patients suffer from heart failure with preserved ejection fraction (HFpEF) [49,50].

Due to the methodology used for heart failure prevalence estimations, unrecognized heart failure patients are not taken into account. Thus, a relevant number of unreported cases is very likely. Within the population of unrecognized cases, up to 76% are considered to be patients with HFpEF [49]. The main reasons for the underestimation of heart failure are the nonspecific symptoms and overlap with other morbidities and the lack of availability of essential diagnostic tests, such as echocardiography, and the measurement of natriuretic peptides in primary care medicine. Using interpolations, heart failure prevalence, including recognized and unrecognized cases, is estimated to be 4.2% within the general population [48].

### 4.2. Utilization of Wearables in Heart Failure

Wearables provide various functional and physiologic data for monitoring heart failure symptoms and status. These data might be used for the management of heart failure patients in addition to data acquired during in-patient and out-patient visits, or by implantable cardiac devices. Wearables offer the opportunity to assess patients’ status closely outside the classic clinical settings. However, most of these devices are developed as a wellness or lifestyle feature rather than medical devices with approval by local authorities. Approval by the FDA or other local authorities is a prerequisite for integration into patient clinical management.

Most wearable technologies currently available depend on a connected smartphone or tablet. However, smartphones are currently used by only 77% of the general population. In the typical heart failure population >65 years of age, only 53% own a smartphone (www.statistica.com (accessed on 2 April 2021)). This fact currently limits the use at least of smartphone-based wearable technologies in the heart failure population. However, even in this age group, smartphone usage is increasing by 7% per year. Thus, in a few years, smartphones are expected to be used in >70–80% of the people in this age group.

### 4.3. Wearables for Detection of Heart Failure in the Community

An additional benefit of the implementation of wearables for the screening of unrecognized heart failure and support of current heart failure management is conceivable. Wearables are able to measure and acquire a number of heart failure surrogates. Heart rate, blood pressure, temperature, body weight changes, step count, posture, activity, lung fluid and heart failure specific questionaries are a selection of parameters acquired by wearables being potentially used for heart failure management (Table 2).

Due to the high estimated number of unreported cases in the general population, contemporary wearables might help to screen for heart failure in the general population. Studies on the use of wearables to identify unrecognized heart failure in the community are currently lacking. In a pilot study, Shah et al. used the PPG and accelerometer data acquired by a wristband wearable in in-patients for the diagnosis of heart failure [51]. They demonstrated that the PPG and accelerometer data had a diagnostic accuracy of 74%. If these data were combined with a selection of patient characteristics, co-morbidities, heart rate and blood pressure, the accuracy increased to 82% [51]. Studies in the community on heart failure screening are generally conceivable. Clinical trials on screening for subclinical AF in the general population have shown that the concept of wearable-based screening for specific diseases is feasible [52], and could, therefore, be translated into a heart failure cohort. As heart failure is a clinical diagnosis, the challenge in screening for heart failure is the identification of asymptomatic individuals. Multiple parameters must be used for screening and diagnosis. Focusing on a population >65 years of age and screening for risk factors of heart failure and co-morbidities might identify heart failure patients and guide further diagnostic evaluation [53,54].

### 4.4. Wearables for Ambulatory Heart Failure Management

Patients with heart failure admitted for heart failure decompensation will have another hospitalization within 30 days in 25% and in 40% of the cases within 12 months after discharge [55]. Each decompensation and hospital admission is associated with higher mortality, and furthermore, heart failure hospitalization relevantly burdens the health care system [56]. Thus, a reduction in heart failure decompensations lowers mortality and reduces the cost burden on the health care system. Studies using telemedicine monitoring weight gain or intrathoracic impedance have failed to reduce hospitalizations. Despite some methodological weaknesses, these studies indicate that the use of these single parameters is probably not sufficient to monitor heart failure status, although thoracic impedance is able to detect cardiac decompensation far upstream in the cascade [57,58,59]. The management of patients with heart failure using direct pulmonary pressure monitoring (CardioMEMS) reduces heart failure hospitalization by 27% [60]. However, this technology is invasive and expensive. In the MULTISENSE trial, heart failure hospitalization could be predicted with a sensitivity of 70% using a number of parameters provided by implantable cardiac electronic devices [61]. From these experiences, the management of chronic heart failure using wearables should ideally use multiple parameters and upstream monitoring of intracardiac diastolic pressure or tissue water [62,63].

The spectrum of wearable devices includes clothing, such as vests, shirts, socks, and accessories, such as smartwatches, wristbands, patches, glasses and rings. These biosensors are able to detect heart failure-specific physiologic parameters. This information can be supplemented by anamnestic information of the patient.

### 4.5. Activity in Heart Failure

Several studies have demonstrated that wristband-, smartwatch- or patch-integrated pedometers or accelerometers are able to provide data on the activity in heart failure patients [64,65,66,67,68]. The accuracy of the step count differs between different devices and manufacturers. Walking speed and the side the watch or wristband is worn impact step count and activity measures [69]. Despite these inaccuracies, step count seems to correlate with the New York Heart Association (NYHA) functional class. In a study comparing patients in NYHA class II and III over a period of 2 weeks, patients in NYHA class III revealed significantly fewer step counts [70].

Activity measured via accelerometers is associated with prognosis. In a Japanese population, a step count of <4889 steps per day was an independent risk factor with a hazard ratio of 2.28 [71]. Loprinzi demonstrated in an American population that a 1-h increase in daily activity lowers the risk of death by 35% [72]. In a small study with 10 patients admitted for newly diagnosed heart failure, activity measured via daily step count correlated with left ventricular ejection fraction (LVEF), six-minute walk test (6MWT) and peak VO2 after hospital discharge (r = 0.44, r = 0.67 and r = 0.57, respectively) [73]. A Czech study demonstrated a decrease in activity during COVID-19 quarantine in heart failure patients. In the first 3 weeks of restrictions, daily step count decreased significantly by 16.2% compared to the period before the beginning of the quarantine [74]. Currently, there is no standard in the measurement of physical activity. Further trials are in progress to compare wearable derived data with classic heart failure measures, such as 6MWT, laboratory and specific heart failure quality of life questionnaires (NCT4191356). A prerequisite for the implementation of activity measure into heart failure management is standardization and prospective clinical trials to prove that the activity-guided management of heart failure has a prognostic impact [75].

### 4.6. Blood Pressure Control in Heart Failure

Recently, a smartwatch-integrated sphygmomanometer was approved by the FDA for wearable-based blood pressure measurement. Kuwabara et al. showed only minimal deviation from manual standard measurements [76]. Wearable-based blood pressure monitoring may support the prevention of heart failure, as hypertension is among the dominant causes of heart failure. In chronic heart failure, close blood pressure measurements may allow the optimal titration of heart failure medication to avoid hypotension and increase therapy adherence.

### 4.7. Heart Rate and Arrhythmia Management

Most wrist-worn wearables or adhesive patches are able to monitor heart rate either via PPG or ECG. PPG measurements are especially vulnerable during exercise. At rest correlation with the standard ECG using different devices was acceptable. However, at different levels of exercise, the limits of agreement were very poor (−22.5 to 26.0 bpm up to −41.0 to 36 bpm) [77]. Moayedi et al. confirmed the limitations of the PPG-based measurement of heart rate in a heart failure population [78]. Due to the vulnerability of the PPG towards motion heart rate, measurement and monitoring should focus on resting heart rate, which shows the best correlation with the ECG gold standard.

### 4.8. Intracardiac Pressures and Tissue Water

Changes in intracardiac pressures and increases in tissue water arise at the beginning of the cascade of cardiac decompensation well before clinical signs emerge. Thus, monitoring of these physiologic parameters is feasible by implantable sensors. Sensor-guided management has been shown to reduce heart failure hospitalization [60]. Noninvasive wearables, such as patches, shirts or vests, are able to detect thoracic fluid accumulation by impedance measurements, remote dielectric sensing (ReDS) or seismocardiography. Feasibility and observational studies could demonstrate that these emerging technologies are able to differentiate between compensated and decompensated heart failure status or even reduce hospitalizations [79,80]. Another method to monitor thoracic fluid is a radiofrequency sensor included in a patch (µCor, Zoll, Pittsburgh, PA, USA). The technologies based on a single parameter to detect heart failure decompensation must prove their clinical value in further clinical trials (NCT03586336 and NCT03476187). In the Link-HF trial, a multisensor approach, including ECG, accelerometer, temperature and intrathoracic impedance, was analyzed [68]. After a normalization period, this multisensor patch was able to show a sensitivity of 76–88% and a specificity of 85% for an alert with a median of 6.5 days before decompensation [68]. The Nanowear wearable Heart Failure Management System Multiple Sensor Algorithm Development and Validation trial is currently investigating the SimpleSENSE device and whether heart failure hospitalization can be predicted by this multisensor technology (NCT03719079). The preliminary clinical data on invasive and noninvasive approaches on thoracic fluid assessment show that the sensitivity of the wearables is comparable to invasive sensors and cardiovascular implantable electronic device-based monitoring [61].

The wearable-based management of heart failure might be a promising addition in the individualized treatment of heart failure. Especially for devices monitoring thoracic fluid or intracardiac pressures, a standard operating procedure for the management of the patient is essential, as the patient is still asymptomatic in this early stage of decompensation. For the implementation of wearable technologies in clinical routine, we need further studies and evidence with a focus on outcome and cost effectiveness.

## 5. Wearables to Assist in Transition of Medical Management from the Clinic to the Home

In recent years, there has been a shift in the direction of de-institutionalization by moving hospital care towards community-based care due in part to a growing elderly population with a greater burden of chronic disease and scarcity of hospital beds. The importance and urgency of this have further been realized with the COVID-19 pandemic, which has led to cancellations of nonurgent hospital visits and hospitalizations. In general, there is now an increased acceptance of a telehealth model by both patients and healthcare providers alike. Nonetheless, among the major challenges of community-based care is the lack of access to information derived from routine examination and monitoring that is otherwise afforded by direct patient contact. In this regard, leveraging data via remote patient monitoring (RPM) from wearable devices may help overcome this obstacle. In the real-world setting, teleconsultations instead of outpatient clinic visits have been made possible by implementing an on-demand app-based heart rate and rhythm monitoring infrastructure [81,82,83].

The use of RPM is not a new concept. The benefit of RPM has been demonstrated in implantable cardiac devices. In fact, the use of RPM in patients with implanted devices (pacemakers, ICDs and cardiac resynchronization therapies) has been shown to be associated with a reduction in mortality, depending on the level of adherence [84,85]. The findings from these studies were confirmed in a meta-analysis of nine randomized controlled trials which also found reduced odds of inappropriate shock with the implementation of RPM [86]. However, there are limited data on the use of wearables in this setting.

Modern wearables are equipped with sensors that are able to monitor physiological parameters, such as temperature, heart rate, HRV, respiratory rate, arterial oxygen saturation, thoracic fluid content and blood pressure. It has been suggested that these wearables may be comparable to implanted devices for the detection of impending heart failure rehospitalization [68]. In such patients, the early detection and institution of treatment may prevent subsequent deterioration. Furthermore, it may have a role in patients with viral-like infections and reduce the spread of outbreaks [87]. A study using retrospective data from the Fitbit wearable found that it could be used to predict influenza-like illness at an individual- and population-level [88]. It has also been tested in patients with the COVID-19 infection, where physiological data derived from this device have been shown to relate to the severity of illness on specific days [89].

**Figure 4:**
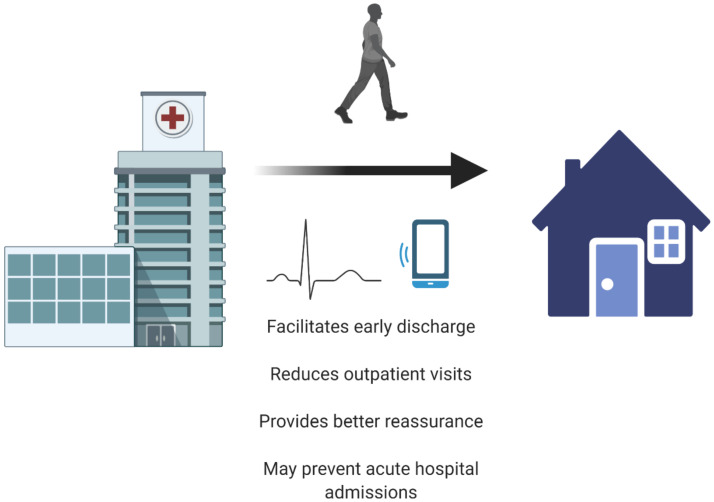
Implementation of wearables may facilitate the transition from hospital-based to home care (created with BioRender.com, accessed on 2 April 2021).

The potential benefits of wearables in community-based care include an improvement in patient outcomes and quality of life, reduced anxiety levels in patients with chronic diseases who are monitored continuously, reduced hospital admissions and visits and decreased length of hospital stays (Figure 4). Furthermore, it facilitates a patient-centered care model with better patient–physician collaboration.

Nevertheless, with the increased adoption of RPM, there needs to be greater data transparency, better protocols for the safe implementation of RPM and more user-friendly systems [90]. Further hardware refinements are also needed to address some of the current challenges faced with the use of wearables for RPM that relate to long-term application (e.g., patches), skin-device interface and energy autonomy [91].

## 6. Wearables for Return to Play in Patients with Congenital and Acquired Cardiovascular Disease

Cardiac deconditioning is often a lifelong issue in children and young adults with congenital heart disease, inherited arrhythmias and cardiomyopathies. These patients often do not have the opportunity, inclination or education to participate in safe and effective exercise, and historically, their physicians and parents have further limited their participation and fostered a fear of sports. This affects their cardiopulmonary health, and has psychosocial consequences, especially when a lack of participation in peer activities or sports leads to isolation and further sedentary behaviors [92]. The past decade has witnessed a paradigm shift in sports allowances in patients with congenital heart disease [93], arrhythmias [94,95], cardiomyopathies [96,97,98] and even ICDs [99,100]. Can direct-to-consumer monitors assist in the present trajectory of increased participation in sports in a population of children, adolescents and young adults with congenital and acquired heart disease, arrhythmia syndromes and cardiomyopathies?

These devices are part of the evolution from episodic to continuous patient care in adult cardiac disease. There are numerous pediatric cardiac conditions that might be better managed with this technology [101]. However, most mobile health technologies, especially wearable biosensors, are not designed for children. As in adults, there are several aspects of disease in this younger population that should be considered of interest: heart rate monitoring, arrhythmia detection, blood pressure monitoring and supporting exercise and rehabilitation. Unlike adults, AF will rarely be the suspected arrhythmia. “Normal” heart rate and blood pressure are age- and occasionally disease-dependent, and even the duration and depth of exercise is age- and disease-dependent.

Children and adolescents with congenital heart disease, inherited arrhythmias and cardiomyopathies often spent a lifetime under the “medical microscope”, with frequent physician visits, procedures, hospitalizations and concerned parental scrutiny. Device design must allow pediatric and adolescent patients to act and feel like kids and teens, not like patients under further surveillance [101]. Parents and children with chronic illnesses often show signs of increased stress, so wearability should not worsen this and should target improved quality of life. It is interesting to note that the use of insulin pumps reduces both patient and caregiver anxiety, in part because of decreased fear of hypoglycemia [102]. Validated wearables should aim to do the same for high-risk congenital and acquired heart disease, cardiomyopathy and inherited arrhythmia populations.

This is a population where hospitalizations, surgeries, medications and procedures are expected and where cardiac rehabilitation, heart rate and rhythm monitoring, response to medications and return to play can be supported by validated wearables. For example, continuous monitors might be useful to up titrate or assess the heart rate, blood pressure and arrhythmia response to beta blocker therapy. Data will provide continuous assessment versus a single snapshot provide by an in-person clinic assessment, ECG and exercise testing.

Children and adolescents with inherited arrhythmia syndromes or channelopathies represent a unique population where this technology has the potential for a number of applications. This is a group of conditions where sports allowances vary but are becoming more permissive, sometimes in contrast to guideline recommendations. The LQTS is the most common channelopathy and the one for which we have the most data about risk and sports participation. Exercise has been associated with arrhythmic events in LQTS patients, especially those with type 1 LQTS [103]. However, many LQTS patients exercise despite the restrictions, and recent data suggest that LQTS subjects who receive appropriate disease-specific therapy can participate in sports safely [94] Current guidelines are more permissive in this population [95]. As the guidelines and cardiologists become more liberal in their approach to sports in this population, patient-friendly monitors could provide a measure of reassurance as this population, previously restricted, engages in sports. Wearable monitors can be used to follow the QT interval at baseline, with the addition of medications, at rest and with activity in patients with LQTS [40]. This could help assess for arrhythmias and worsening of repolarization with more profound QT prolongation or the appearance of T wave alternans that might predict arrhythmias.

Hypertrophic cardiomyopathy (HCM), the most commonly inherited cardiomyopathy, has been linked to sudden death during exercise [104]. Traditionally, this has been a population with significant sports restrictions. However, there is a move to liberalize restrictions as researchers and cardiologists challenge the widely held view that HCM is the most common cause of sudden cardiac death in young patients who participate in vigorous activities [96]. There is a prospective observational study (Lifestyle and Exercise in HCM) enrolling HCM patients across a range of activity levels comparing outcomes in those participating in high levels of activity with more sedentary individuals. Data from wearables will likely prove to be of great utility in this population as we find a balance between sports and safety and finally allow this population the physical and psychological benefits of an active lifestyle.

Acquired heart disease in children and adolescents is far less common than in adults. Acquired heart diseases in this young population where a safe return to sports must be considered include Kawasaki disease, rheumatic carditis and viral myocarditis. The recent COVID-19 pandemic has resulted in two new potential acquired cardiac diseases in young people: myocarditis as a result of the acute infection and heart failure/myocarditis as a consequence of the multisystem inflammatory syndrome in children [105]. Significant cardiac morbidity has been observed among hospitalized COVID-19 adult patients occurring in 22%, higher than the 1% in non-COVID-19 acute viral infections [106,107]. Myocarditis from myocyte invasion by the virus can result in dysfunction, arrhythmias and death. Return to play is predicated on the normalization of ventricular function, absence of biomarkers of inflammation and necrosis and absence of arrhythmias [107]. Risk assessment after recovery at present is based on extensive cardiac testing with echocardiograms, stress testing and rhythm monitoring. Wearable monitors in this scenario will enable the continual assessment of unlimited duration, not a single snapshot in time. The editorials on this topic acknowledge the many uncertainties about this disease and its recovery [107].

Successfully integrating continuous monitoring into cardiovascular care as patients increase sports engagement and return to sports after an event or illness will require collaboration between clinicians, industry and regulatory bodies. Small children will be uniquely challenging with size, physiologic and compliance considerations, but teens and young adults will be an “easy sell” with their technological sophistication and openness to adopting and using new technologies. The challenges that exist can and will be solved. This new technology is poised to extend the physician’s ability to safely care for young patients, some who are adopting a more active lifestyle and others reengaging in sports after an illness.

## 7. Sleep Apnea

Sleep-disordered breathing is common in the community, and the prevalence is increasing. Current estimates report that the prevalence of moderate-to-severe sleep-disordered breathing (apnea-hypopnea index, measured as events/hour, ≥15) is 10% among 30–49-year-old men, 17% among 50–70-year-old men, 3% among 30–49-year-old women and 9% among 50–70 year-old women, all with relative increases of 14–55% over the past 2 decades [108]. Sleep apnea, through multiple mechanistic pathways, is associated with an increased risk of hypertension, arrhythmias, stroke, heart failure and cardiovascular disease [109].

Screening for sleep apnea is typically through continuous nocturnal oximetry, and multiple oximetry tools are available by prescription and direct-to-consumer. However, although oximetry is used for sleep apnea screening, polysomnography remains the gold standard for the diagnosis. A study using a wrist-worn reflective photoplethysmography was compared to polysomnography in 188 recordings with a favorable correlation (correlation = 0.61; estimation error = 3 ± 10 events/h) [110]. The estimated apnea–hypopnea index correlation was also favorable compared to polysomnography across different severities of obstructive sleep apnea (ROC–AUC: mild: 0.84; moderate: 0.86; severe 0.85). As noted by the authors of this study, the wrist-worn tool diagnostics can be implemented in wearables, such as smartwatches.

Other applications of electrogram monitoring may also be relevant to the diagnosis of sleep apnea, in isolation or combination with photoplethysmography, as the disorder is often characterized by paroxysms of nocturnal bradycardia and conduction delay as well as intermittent tachyarrhythmias. Sleep apnea can also impact the neuromodulation of the heart, which influences HRV [111]. Using HRV in 30 patients with sleep apnea, the RR interval correlated with the apnea hypopnea index (r = 0.663, *p* = 0.003). Using the RR interval alone, 25 of the patients would have been diagnosed with obstructive sleep apnea.

## 8. Artificial Intelligence

Among the most straightforward applications of AI to wearable devices is the automatic interpretation of ECG signals and the classification of arrhythmias from wearable devices [112]. For example, AliveCor obtained FDA approval for a suite of AI algorithms to detect not only AF but also sinus rhythm with premature ventricular contractions, supraventricular ectopy and sinus rhythm with wide QRS [113]. Such AI algorithms could alleviate the clinicians’ burden in manually interpreting the increasing number of ECGs obtained from wearable devices and only generate alerts when a clinical action is needed.

AI applied to ECGs could also help identify other cardiac conditions beyond arrhythmias. Many cardiovascular diseases, such as left ventricular systolic dysfunction (LVSD) and HCM, are underdiagnosed, because the diagnostic tests are too costly to perform in a broad asymptomatic population. In the past, statistical models were developed based on discrete ECG features, such as left bundle branch block and ST-T segment changes [114,115,116]. However, such models were based on clinicians’ identification of these features, thereby requiring manual ECG interpretation. Additionally, traditional statistical models, e.g., logistic regression models based on a few ECG features, had poor performance, resulting in limited clinical utility.

In recent years, a number of ECG-based AI algorithms have shown great performance in detecting cardiac conditions, such as LVSD [117,118], HCM [119] and MI [120]. The performance of these AI algorithms is typically better than risk scores currently used in routine practice, e.g., the CHA_2_DS_2_-VASc score. For example, one ECG-based deep learning algorithm showed a c statistic of 0.93 for identifying low ejection fraction [117,118], and another algorithm identified HCM with a c statistic of 0.96 [119].

When assessed in a pragmatic randomized controlled trial, the AI ECG algorithm, serving as a clinical decision support tool, significantly improved the diagnosis of low EF in 22,641 patients managed in routine primary care settings [121,122]. Therefore, the AI tool could identify asymptomatic LVSD at the early stage, so the treatment can be initiated to prevent HF progression and mortality [123,124] When applied in the emergency department, the ECG-based AI algorithm outperformed NT-proBNP for identifying patients with LVSD, which could serve as a quick, inexpensive approach to guide subsequent echocardiography decisions in acute settings [125].

As an increasing number of consumer-grade wearables are equipped with the capacity to record ECGs, the ECG-based AI algorithms can help screen broad populations for rare conditions, such as HCM, asymptomatic LV dysfunction or occult arrhythmias. The ability to detect MI and LVSD could also alert patients to seek medical care early and inform diagnostic approaches when patients arrive in the hospital. However, a major challenge is that most of the AI algorithms have been trained based on using signals from conventional 12-lead ECGs. As such, the algorithm performance when using single-lead ECGs is variable, and the algorithms may require retraining or tuning before they can be readily applied to ECGs obtained from wearable devices.

ECG-based algorithms can also play a role in chronic disease management, such as hypertension and diabetes [126,127]. For example, an AI algorithm was developed based on raw ECG signals recorded by a commercial wearable device (Medtronic Zephyr BioPatch™ HP80) and has been shown to predict hypoglycemic events [127]. Although commercial devices are available for patients to monitor HbA1c at home, blood glucose testing requires a drop of blood from a finger prick and does not allow continuous monitoring, so continuous monitoring using AI-enhanced wearable devices is an attractive alternative.

The data collected from wearable devices could also help augment existing prediction models. Most prediction models are developed using data collected from clinical settings and lack information, such as lifestyle factors, sleep patterns and daily activities, which are known risk factors for cardiovascular diseases and mortality [128,129]. Traditionally, such data are collected based on self-reporting, and are thereby subject to recall bias. Recently, a number of studies assessed the relationship between health outcomes and data collected from wearable devices, such as step count, sleep duration and sleep variability [130,131,132]. For example, one study found that a higher step count on postoperative day one was associated with significantly lower odds of prolonged length of stay in patients undergoing major surgery [131]. Therefore, AI models could be more powerful when data from wearable devices are integrated with clinical data in the electronic health record.

Historically, the derivation and application of machine learning algorithms have typically been computationally expensive and performed by supercomputers or high-end workstations. Recently, cloud services, such as Amazon AWS or Google Cloud, have been increasingly used for data storage and maintenance, given the reduction in cost and maintenance as well as the elasticity that cloud architectures provide [133]. Previous studies have demonstrated the utility of high-performance cloud computing resources, directly integrated into the EHR, for developing and deploying advanced predictive models [134]. Therefore, AI could be embedded into EHR, leveraging medical history as well as real-time data transmitted from the wearable devices, to generate clinically actionable alerts. However, future studies are needed to prove this concept. Additionally, some “lean”/less computationally demanding ML algorithms, particularly those that are applied to shorter times series data, can be applied directly on wearable devices without the must move data to a cloud service.

The real-world effectiveness and clinical impact of AI applied to wearable devices also depends on clinicians’ and patients’ uptake. Although many clinicians are interested in new technologies, others may be skeptical. AI is often considered a “black-box” technology, making it difficult for some clinicians to trust the results or communicate the meaning of AI-derived findings to patients [135,136]. Moreover, a recent study of abnormal pulse detected by the Apple Watch found that nearly 90% of the alerts did not lead to clinically actionable diagnoses, leading to concerns of increased low-value health utilization [137].

A recent study of patients’ views of wearable devices and AI found that only half of patients feel that the digital tools and AI constitute an important opportunity, and 11% considered them a danger [138] Some patients fear that the misuse of technology could threaten the humanistic aspect of health care, which is a valid concern, since the use of wearable devices is often outside the health system without any interaction with their clinicians. This could contribute to the high dropout and incomplete follow up of some large-scale implementations of digital monitoring programs and highlight the need for patient engagement and education [139,140].

In summary, AI applied to wearable devices could enable the automatic classification of arrhythmias, early detection of other cardiac conditions, improved management of chronic diseases and augmented performance of existing prediction models. However, there are a number of challenges, ranging from the transportability of the algorithms, and the integration of data from wearable devices with clinical data, to the adoption by clinicians and patients. How to maximize the potential of AI and wearable devices without significantly increasing health utilization and burden on clinicians and patients remains to be further investigated.

## 9. Future Perspectives

Mobile technologies are growing rapidly and will be ubiquitous in the near future. Supporting evidence of the benefit for users, patients or doctors is crucial for future use in clinical practice. Unselected measurements of singular surrogates without predefined decision pathways have not turned out to be successful, despite promising concepts [58]. Appropriate clinical pathways, therefore, must be developed and evaluated measuring various parameters or surrogates using wearables in order to provide a benefit in different clinical scenarios or patient cohorts. This also includes appropriate training for users, patients and prescribing physicians.

One concern among doctors when using wearables and new technologies is data overload [141]. With the expected amount of data, AI can help with the selection and presorting and make the evaluation more efficient. AI can also unlock completely new and previously unused data. The measurement of vocal biomarkers shows a good correlation with the degree of pulmonary hypertension [142]. In patients with heart failure, vocal biomarkers correlate with heart failure hospitalizations and mortality [143]. In a proof-of-concept study, AI was able to ensure the contactless detection of cardiovascular arrests using smart speakers or smartphones and differentiate between agonal breathing and hypopnea, central apnea and obstructive apnea [144].

These early results are suggestive of a future role for vocal biomarkers in remote monitoring and remote patient management.

Since the COVID-19 pandemic, the acceptance and implementation of mobile technologies was catalyzed in the field of cardiac electrophysiology, as it was in every field of medicine [145]. The experience of the TeleCheck-AF project shows that a fast, efficient and powerful implementation of new clinical workflows using mHealth is feasible [83]. The integration of wearable technologies in other subspecialties of cardiology should take into account the different perspectives of all players in this field, namely, the user or patient and the treating physician, but also the legislation, insurance and provider.

## 10. Conclusions

Advances in wearable technologies have provided new opportunities for the diagnosis and management of cardiovascular diseases and their risk factors. The possibilities and implementation of wearable cardiac monitoring beyond AF are increasing continuously. The management of nonatrial fibrillation arrhythmias represents a broad field of wearable technologies in cardiology. Implementation in other patient cohorts, such as STEMI, heart failure or sleep apnea, is feasible and expanding. Medical devices that are marketed directly to consumers, in addition to current and future physician-ordered testing devices, will augment our current reach and effort in the management to help address the increasing worldwide burden of cardiovascular diseases. In addition to appropriate accuracy, clinical studies must address the validation of clinical pathways, including the appropriate device and the clinical decisions resulting from the surrogate assessed. These tools must lead to actionable approaches to reduce cardiovascular disease risk factors or augment the use of guideline-based therapies. As each technology is considered, its use must be weighed against the impact of the demand on the healthcare system for the incorporation and interpretation of the diagnostic data, the value of downstream diagnostics and therapies that will naturally increase with broader screening, and the personal consumers of these technologies as they are drawn into a more dynamic relationship with their health, physiology and healthcare providers.

## Figures and Tables

**Table 1:** Types of cardiac monitoring.

Device	Maximum Duration of Monitoring	Continuous Recording	Event Recording	Features
12-lead ECG	Single timepoint	No	No	Easy and cheap method of monitoring; good diagnostic capability if captures arrhythmia
Holter monitoring	72 h	Yes	Yes	Short-term but quantifies burden of arrhythmia
Patch monitor	1–3 weeks	Yes	Yes	Intermediate duration with straightforward application and improved patient compliance
External loop recorder	1 month	No	Yes	Provides long-term monitoring of rare, sustained events
Smartphone monitor	Indefinite	No	Yes	Available direct to consumer; inexpensive; provides long-term monitoring of rare, sustained events; requires a Smartphone
Mobile cardiac telemetry	1 month	Yes	Yes	Real-time cardiac monitoring with remote capability; relatively expensive
Implantable loop recorder	3 years	Yes	Yes	Provides long-term monitoring of arrhythmias; requires invasive procedure; relatively expensive
Pacemaker or ICD	Indefinite (with box changes)	Yes	Yes	Long-term monitoring with remote capability; able to deliver therapy in certain situations; requires invasive procedure; expensive
WCD	Indefinite	Yes	Yes	Very expensive; able to deliver therapy for life-threatening ventricular arrhythmias; dependent on compliance
Smart textile-based garments	Indefinite	Yes	Potentially	Rapidly developing field that is yet to be established

ECG, electrocardiogram; ICD, implantable cardioverter-defibrillator; WCD, wearable cardioverter-defibrillator.

**Table 2:** Surrogates for wearable measurement in heart failure.

HF Parameter	Method	Device
Thoracic fluid	Thorax impedance measurement, remote dielectric sensing, seismocardiography	Patch, vest, smart shirt
Activity	Pedometers, accelerometers,	Patch, wrist band, smart watch, smart shirt
Blood pressure	Sphygmomanometer	Smart watch, wristband
Body Weight	Body weight measurement	Smart socks, scale
NYHA functional class	Questionnaires, applications	Smart phone, tablet
QoL	Questionnaires, applications	Smart phone, tablet
Heart rate, heart rhythm	ECG, PPG	Smart watch, wrist band, patch, chest band, smart shirt

ECG, electrocardiogram; HF, heart failure; NYHA, New York Heart Association; PPG, photoplethysmography; QoL, quality of life.

## Data Availability

Not applicable.
